# Psychosocial treatment outcomes of common mental disorders vary widely in persons in low- and middle-income countries affected by humanitarian crises and refugees in high-income countries

**DOI:** 10.1192/bjo.2022.73

**Published:** 2022-06-01

**Authors:** Alvin Kuowei Tay, Jessica Carlsson

**Affiliations:** Discipline of Psychiatry and Mental Health, School of Clinical Medicine, University of New South Wales, Australia; Competence Centre for Transcultural Psychiatry, Mental Health Centre Ballerup, Mental Health Services of the Capital Region of Denmark, Denmark; and Department of Clinical Medicine, University of Copenhagen, Denmark

**Keywords:** Refugee, psychosocial interventions, treatment outcome, trauma, mental health

## Abstract

This commentary discusses methodological and contextual factors that might account for variations in psychosocial treatment outcomes found in persons in low- and middle-income countries affected by humanitarian crises and refugees. Factors discussed are related to cultural adaptations, content and intensity of treatment, population characteristics and factors related to research design.

A significant development in global mental health is the emergence of psychosocial interventions for common mental disorders (CMDs), including depression, anxiety and post-traumatic stress disorder (PTSD). Yet, there remain substantial variations in mental health outcomes across intervention studies undertaken with refugee, forcibly displaced and conflict-affected populations from diverse settings.

**Figure:**
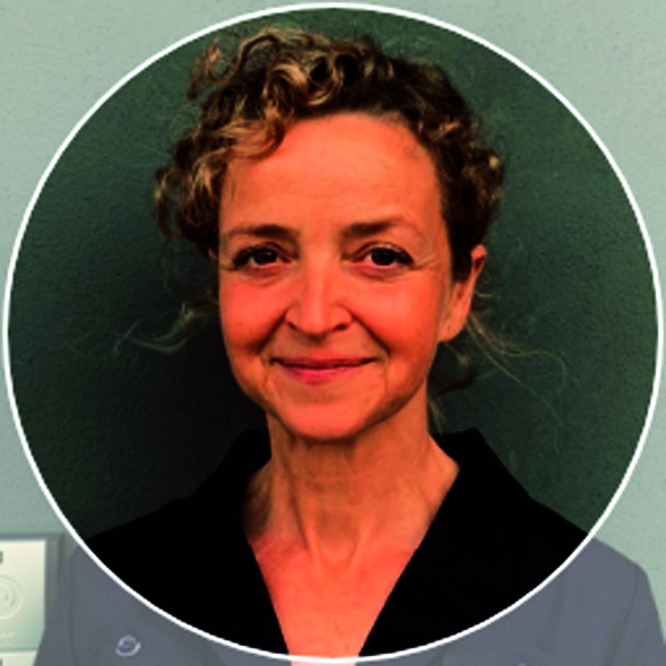

**Figure:**
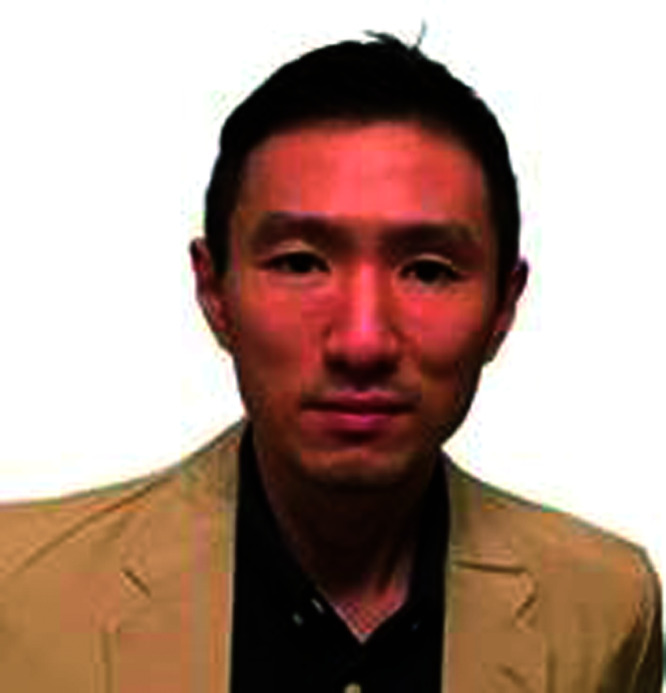

This commentary first synthesises the current evidence for psychosocial interventions, drawing on systematic reviews, meta-analyses and key studies with refugees and displaced and conflict-affected persons living in low- and middle-income countries (LMICs) (including humanitarian settings), and refugees resettled in high-income countries (HICs). We excluded migrants of different types and the general populations living in LMICs and HICs. We then outline methodological and contextual factors that might explain the differential outcomes of depression, anxiety and PTSD (referred to as CMDs). We apply the definition of psychosocial interventions provided by the US Institute of Medicine, which includes ‘interpersonal or information activities, techniques or strategies that target biological, behavioural, cognitive, emotional, interpersonal, social or environmental factors with the goal of reducing symptoms of these disorders and improving functioning or well-being’.^1^ For comparison purposes, we draw on the reported outcome data (pre–post changes in CMD symptoms) from current studies based on effect sizes and adjusted mean differences between treatment arms.

Evidence suggests that, compared with primarily inactive controls, psychosocial interventions effectively treat symptoms of CMDs in refugees and displaced persons living in LMICs and HICs.^2–5^ Within LMICs (including humanitarian settings), the most extensive evidence supports cognitive–behavioural therapy and interpersonal psychotherapy.^5^ In this context, the evidence supports the implementation of task-shifted psychosocial interventions delivered by non-specialists.^6^ Psychosocial interventions that are culturally and contextually adapted generally have produced the most robust evidence. Within the humanitarian contexts in LMICs, however, there are very few psychosocial interventions that are evaluated and systematically implemented in local health systems.^7^ Within HICs, narrative exposure therapy offers the most robust evidence for PTSD among resettled refugees.^2,4^

Although the cumulative body of systematic reviews and meta-analyses is supportive of psychosocial interventions mitigating CMDs, the overall effect sizes vary substantially across studies with refugees who relocated to HICs, and refugees and displaced and conflict-affected persons living in LMICs.^4,8^ Specifically, there is significant heterogeneity in the CMD outcomes across studies and settings, with varying levels of strength (of association) and credibility (of evidence) in the outcomes reported. These variations may be attributed to a combination of a wide range of methodological and contextual factors.

Evident in the current systematic reviews and meta-analyses are the significant inter-study differences in treatment content, i.e. the type of psychotherapeutic techniques or strategies. Further, the length of the treatment course, sessions and intensity, including the ‘dosage’ of each psychotherapeutic technique, vary considerably. Particularly, the duration of treatment varies across settings from three to 17 or more sessions, with interventions in HICs typically lasting over 4 months on average.^4^ A growing body of research has found positive effects of brief interventions, ranging from five to seven sessions, on CMD outcomes in LMICs and humanitarian settings. Because of their brevity, ease of application, effectiveness and scalability, these so-called ‘scalable’ or ‘low-intensity’ interventions are now being scaled up for refugees in HICs and LMICs,^9^ although the effect sizes again vary considerably across contexts.^5^ Compared with longer psychotherapeutic treatments, shorter treatments appear to be less effective, although they are generally superior to waitlist or treatment-as-usual controls.^10^

Further, there is no consistency in the ‘dose’ of individual elements of each psychosocial intervention applied across studies, with more flexibility in some interventions allocating more time, sessions and salience to specific strategies tailored to the individual's clinical profile or presentation.^11^ By contrast, other interventions follow a more prescriptive approach, requiring a set number of sessions for each treatment component.^12^ Other issues of interest, although not often described in detail, are to what degree and how psychotherapeutic interventions and measurement tools have been adapted to the culture and context of the study population. The use of different measures, ranging from self-report questionnaires and symptom checklists to diagnostic interviews, with varying degrees of cultural and contextual adaptation, are likely to contribute to discrepancies in outcomes across psychotherapeutic interventions. There is a need to consider the cultural and contextual adaptation of measures across different settings.^13^

There are inherent distinctions in mental health and psychosocial support (MHPSS) services offered in HICs and LMICs, in that specialised MHPSS services are generally available for refugees in the former setting. In contrast, MHPSS services are typically task-shifted to non-specialists in LMICs.^14^ A high proportion of studies are conducted by the originators of the method or their direct trainees, and are at risk of allegiance bias.^4,5^

There are wide variations in treatment providers across settings. Most treatments in HICs are provided by psychologists, psychiatrists and psychotherapists, through interpreters.^4^ By contrast, in LMICs, MHPSS interventions are task-shifted to, for example, lay counsellors, community health workers or peer helpers, who are familiar with the culture, language and are often trained in evidence-based practices in their native languages.^15^

In addition to the abovementioned factors, key predictors of treatment response in refugee populations that warrant attention are gender, age, time since arrival, premigration trauma, access to support and resources, residency status, access to employment, social relations, baseline symptom severity, length of functional impairment, comorbidities and chronic pain.^16–18^ None of these predictors have been consistently replicated across studies and settings, but should be considered in future studies.

Elucidating key differences in individual and subgroup characteristics in refugees is critical because it helps advance our understanding of how variations in risk profiles influence treatment response in psychosocial intervention studies. Also, more ‘unpacking’ studies are needed to reveal the underlying mechanisms of change that contribute to clinically meaningful change in interventions with refugees. Component analyses^19^ of existing psychotherapeutic treatments will help disaggregate the most effective ‘ingredients’, and incorporate these strategies into a more tailored approach. For example, although trauma-focused cognitive–behavioural therapies are well-supported by current evidence, there have been concerns about the uniform application of elements of exposure to the complexities of refugee trauma, and whether it is necessary for clinically meaningful change in PTSD in trauma-affected refugees.^20^

Systemising which and the way treatment predictors, postmigration stress, displacement stress and psychosocial support systems are measured in intervention studies with refugees will build the evidence for a tailored treatment approach in this population.

Related to some of these population characteristics, the differences in both sampling and referral pathways can influence treatment outcomes. Between studies, there are significant differences in severity and complexity of participants: at one extreme, participants are drawn directly from the community and have had no prior interventions (with less complex and chronic presentations); at the other extreme, participants are referred to specialist clinics (mainly in HICs), often after a range of prior interventions. Therefore, the latter group is more likely to have been ‘selected’ and have treatment-resistant disorders.

This commentary outlines challenges in mental health and psychosocial interventions for refugee, displaced and conflict-affected populations in LMICs and HICs. It is timely to reflect on these critical issues, and chart the steps forward as the research area matures.
